# Detection of a mosaic *CDKL5* deletion and inversion by optical genome mapping ends an exhaustive diagnostic odyssey

**DOI:** 10.1002/mgg3.1665

**Published:** 2021-05-06

**Authors:** Heidi Cope, Hayk Barseghyan, Surajit Bhattacharya, Yulong Fu, Nicole Hoppman, Cherisse Marcou, Nicole Walley, Catherine Rehder, Kristen Deak, Anna Alkelai, Undiagnosed Diseases Network, Maria T Acosta, Margaret Adam, David R Adams, Pankaj B Agrawal, Mercedes E Alejandro, Justin Alvey, Laura Amendola, Ashley Andrews, Euan A Ashley, Mahshid S. Azamian, Carlos A. Bacino, Guney Bademci, Eva Baker, Ashok Balasubramanyam, Dustin Baldridge, Jim Bale, Michael Bamshad, Deborah Barbouth, Pinar Bayrak‐Toydemir, Anita Beck, Alan H. Beggs, Edward Behrens, Gill Bejerano, Jimmy Bennet, Beverly Berg‐Rood, Jonathan A. Bernstein, Gerard T. Berry, Anna Bican, Stephanie Bivona, Elizabeth Blue, John Bohnsack, Carsten Bonnenmann, Devon Bonner, Lorenzo Botto, Brenna Boyd, Lauren C. Briere, Elly Brokamp, Gabrielle Brown, Elizabeth A. Burke, Lindsay C. Burrage, Manish J. Butte, Peter Byers, William E. Byrd, John Carey, Olveen Carrasquillo, Ta Chen Peter Chang, Sirisak Chanprasert, Hsiao‐Tuan Chao, Gary D. Clark, Terra R. Coakley, Laurel A. Cobban, Joy D. Cogan, Matthew Coggins, F. Sessions Cole, Heather A. Colley, Cynthia M. Cooper, William J. Craigen, Andrew B. Crouse, Michael Cunningham, Precilla D'Souza, Hongzheng Dai, Surendra Dasari, Joie Davis, Jyoti G. Dayal, Matthew Deardorff, Esteban C. Dell'Angelica, Shweta U. Dhar, Katrina Dipple, Daniel Doherty, Naghmeh Dorrani, Argenia L. Doss, Emilie D. Douine, David D. Draper, Laura Duncan, Dawn Earl, David J. Eckstein, Lisa T. Emrick, Christine M. Eng, Cecilia Esteves, Marni Falk, Liliana Fernandez, Carlos Ferreira, Elizabeth L. Fieg, Laurie C. Findley, Paul G. Fisher, Brent L. Fogel, Irman Forghani, Laure Fresard, William A. Gahl, Ian Glass, Bernadette Gochuico, Rena A. Godfrey, Katie Golden‐Grant, Alica M. Goldman, Madison P Goldrich, David B. Goldstein, Alana Grajewski, Catherine A. Groden, Irma Gutierrez, Sihoun Hahn, Rizwan Hamid, Neil A. Hanchard, Kelly Hassey, Nichole Hayes, Frances High, Anne Hing, Fuki M. Hisama, Ingrid A. Holm, Jason Hom, Martha Horike‐Pyne, Alden Huang, Yong Huang, Laryssa Huryn, Rosario Isasi, Fariha Jamal, Gail P. Jarvik, Jeffrey Jarvik, Suman Jayadev, Lefkothea Karaviti, Jennifer Kennedy, Dana Kiley, Isaac S. Kohane, Jennefer N. Kohler, Deborah Krakow, Donna M. Krasnewich, Elijah Kravets, Susan Korrick, Mary Koziura, Joel B. Krier, Seema R. Lalani, Byron Lam, Christina Lam, Grace L. LaMoure, Brendan C. Lanpher, Ian R. Lanza, Lea Latham, Kimberly LeBlanc, Brendan H. Lee, Hane Lee, Roy Levitt, Richard A. Lewis, Sharyn A. Lincoln, Pengfei Liu, Nicola Longo, Sandra K. Loo, Joseph Loscalzo, Richard L. Maas, John MacDowall, Ellen F. Macnamara, Calum A. MacRae, Valerie V. Maduro, Marta M. Majcherska, Bryan C. Mak, May Christine V. Malicdan, Laura A. Mamounas, Teri A. Manolio, Rong Mao, Kenneth Maravilla, Thomas C. Markello, Ronit Marom, Gabor Marth, Beth A. Martin, Martin G. Martin, Julian A. Martínez‐Agosto, Shruti Marwaha, Jacob McCauley, Allyn McConkie‐Rosell, Colleen E. McCormack, Alexa T. McCray, Elisabeth McGee, Heather Mefford, J. Lawrence Merritt, Matthew Might, Ghayda Mirzaa, Eva Morava, Paolo M. Moretti, Deborah Mosbrook‐Davis, John J. Mulvihill, David R. Murdock, Anna Nagy, Mariko Nakano‐Okuno, Avi Nath, Stan F. Nelson, John H Newman, Sarah K. Nicholas, Deborah Nickerson, Shirley Nieves‐Rodriguez, Donna Novacic, Devin Oglesbee, James P. Orengo, Laura Pace, Stephen Pak, J. Carl Pallais, Christina G. S. Palmer, Jeanette C. Papp, Neil H. Parker, Jennifer E. Posey, Lorraine Potocki, Bradley Power, Barbara N. Pusey, Aaron Quinlan, Wendy Raskind, Archana N. Raja, Deepak A. Rao, Genecee Renteria, Chloe M. Reuter, Lynette Rives, Amy K. Robertson, Lance H. Rodan, Jill A. Rosenfeld, Natalie Rosenwasser, Francis Rossignol, Maura Ruzhnikov, Ralph Sacco, Jacinda B. Sampson, Susan L. Samson, Mario Saporta, C. Ron Scott, Judy Schaechter, Timothy Schedl, Kelly Schoch, Daryl A. Scott, Jimann Shin, Rebecca Signer, Edwin K. Silverman, Janet S. Sinsheimer, Kathy Sisco, Edward C. Smith, Kevin S. Smith, Emily Solem, Lilianna Solnica‐Krezel, Ben Solomon, Rebecca C. Spillmann, Joan M. Stoler, Jennifer A. Sullivan, Kathleen Sullivan, Angela Sun, Shirley Sutton, David A. Sweetser, Virginia Sybert, Holly K. Tabor, Amelia L. M. Tan, Queenie K.‐G. Tan, Mustafa Tekin, Fred Telischi, Willa Thorson, Audrey Thurm, Cynthia J. Tifft, Camilo Toro, Alyssa A. Tran, Brianna M. Tucker, Tiina K. Urv, Adeline Vanderver, Matt Velinder, Dave Viskochil, Tiphanie P. Vogel, Colleen E. Wahl, Stephanie Wallace, Chris A. Walsh, Melissa Walker, Jennifer Wambach, Jijun Wan, Lee‐kai Wang, Michael F. Wangler, Patricia A. Ward, Daniel Wegner, Mark Wener, Tara Wenger, Katherine Wesseling Perry, Monte Westerfield, Matthew T. Wheeler, Jordan Whitlock, Lynne A. Wolfe, Jeremy D. Woods, Shinya Yamamoto, John Yang, Muhammad Yousef, Diane B. Zastrow, Wadih Zein, Chunli Zhao, Stephan Zuchner, Eric Vilain, Vandana Shashi, John A. Phillips

**Affiliations:** ^1^ Division of Medical Genetics Department of Pediatrics Duke University Medical Center Durham NC USA; ^2^ Center for Genetic Medicine Research Children’s National Hospital Washington DC USA; ^3^ Department of genomics and Precision Medicine School of Medicine and Health Sciences George Washington University Washington DC USA; ^4^ Bionano Genomics Inc San Diego CA USA; ^5^ Division of Laboratory Genetics and Genomics Department of Laboratory Medicine and Pathology Mayo Clinic Rochester MN USA; ^6^ Department of Pathology Duke University Medical Center Durham NC USA; ^7^ Institute for Genomic Medicine Columbia University Medical Center New York NY USA

**Keywords:** copy number variants, epilepsy, mosaicism, optical genome mapping, structural variants

## Abstract

**Background:**

Currently available structural variant (SV) detection methods do not span the complete spectrum of disease‐causing SVs. Optical genome mapping (OGM), an emerging technology with the potential to resolve diagnostic dilemmas, was performed to investigate clinically‐relevant SVs in a 4‐year‐old male with an epileptic encephalopathy of undiagnosed molecular origin.

**Methods:**

OGM was utilized to image long, megabase‐size DNA molecules, fluorescently labeled at specific sequence motifs throughout the genome with high sensitivity for detection of SVs greater than 500 bp in size. OGM results were confirmed in a CLIA‐certified laboratory via mate‐pair sequencing.

**Results:**

OGM identified a mosaic, *de novo* 90 kb deletion and inversion on the X chromosome disrupting the *CDKL5* gene. Detection of the mosaic deletion, which had been previously undetected by chromosomal microarray, an infantile epilepsy panel including exon‐level microarray for *CDKL5*, exome sequencing as well as genome sequencing, resulted in a diagnosis of X‐linked dominant early infantile epileptic encephalopathy‐2.

**Conclusion:**

OGM affords an effective technology for the detection of SVs, especially those that are mosaic, since these remain difficult to detect with current NGS technologies and with conventional chromosomal microarrays. Further research in undiagnosed populations with OGM is warranted.

## Graphical abstract

In a participant with an epileptic encephalopathy of undiagnosed molecular origin, optical genome mapping (OGM), identified a mosaic, de novo 90 kb deletion on the X chromosome disrupting the *CDKL5* gene. Detection of the mosaic deletion, which had been previously undetected by chromosomal microarray, an infantile epilepsy panel including exon‐level microarray for *CDKL5*, exome sequencing as well as genome sequencing, resulted in a diagnosis of X‐linked dominant early infantile epileptic encephalopathy‐2. OGM affords an effective technology for detection of SVs, especially those that are mosaic, since these remain difficult to detect with current NGS technologies and with conventional chromosomal microarrays.

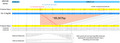

## 1 INTRODUCTION

Structural variants (SVs) are segments of DNA at least 50 bp in length which encompass both unbalanced copy number variants (CNVs) and balanced events such as balanced translocations and inversions (Eichler, 2019). CNVs are not as numerous within the genome as single nucleotide variants (SNVs), but are a significant contributor to genetic disease pathology. Intragenic CNVs account for approximately 10% of disease‐causing variants in Mendelian diseases (Truty et al., 2019). Additionally, larger CNVs that contain multiple genes are known to underlie 10–15% of genetic disorders (Miller et al., 2010). The contribution of structurally balanced SVs to Mendelian disease is less clear. In clinical genetics practice, CNVs are typically interrogated through chromosome analysis, whole‐genome chromosomal microarray analysis (CMA), gene‐specific exon‐level arrays, and CNV analysis of exome sequencing (ES) data, each of which has distinct variant detection capabilities and limitations (Aradhya et al., 2012; Mason‐Suares et al., 2013; Tan et al., 2014). Sensitivity in detecting CNVs varies by methodology, but is never 100% and sometimes is significantly less. For example, the estimated sensitivity for CNV detection utilizing one ES‐based CNV caller was ~76% in comparison to CMA (Krumm, et al. (2012). Short‐read genome sequencing (GS), an emerging technology in clinical practice, has improved sensitivity over ES in detecting CNVs below the level of resolution detectable by CMA, but nevertheless fails to capture the complete spectrum of disease‐causing CNVs, particularly in repetitive regions of the genome (Chaisson et al., 2019; Gross et al. 2019). There is thus a need for more comprehensive detection of CNVs.

Optical genome mapping (OGM) developed by Bionano Genomics is a novel technology with high sensitivity for the detection of SVs ranging from 500 bp to aneuploidies (Bocklandt et al., 2019; Mak et al., 2016; Mantere et al., 2019). While not yet widely available in clinical diagnostics, proof‐of‐principle studies have successfully utilized OGM to detect pathogenic SVs in individuals with known chromosomal aberrations, hereditary breast cancer, DMD and FSHD1 (Barseghyan et al., 2017; Du et al., 2018; Mantere et al., 2019; Zhang et al., 2019). OGM is in the initial stages of being applied to undiagnosed individuals, as presumably there are pathogenic SVs that remain elusive to current clinical SV detection methods (Shieh, 2020).

The Undiagnosed Diseases Network (UDN), a nationwide research study funded by the National Institutes of Health Common Fund, aims to discover diagnoses for the most difficult‐to‐diagnose individuals (including many with non‐diagnostic CMA and ES) through collaborative science and adoption of novel diagnostic methods (Gahl et al., 2016). The UDN has achieved a 33% network‐wide diagnostic rate utilizing genomic sequencing in combination with model organisms (Bellen et al., 2019; Harnish et al., 2019; Wangler et al., 2017), innovative computational approaches (Deisseroth et al., 2018; Holt et al., 2019; Mohanty et al., 2019; Shashi et al., 2019; Wang et al., 2017) and advanced technologies such as transcriptome sequencing (Fresard et al., 2019; Lee et al., 2020). We now report the first successful diagnosis of a UDN participant with optical genome mapping, following extensive prior non‐diagnostic genetic and genomic investigations.

## 2 MATERIALS AND METHODS

OGM (Bionano Genomics, Saphyr System) was performed to investigate clinically‐relevant SVs in a 4‐year‐old male with an epileptic encephalopathy of the undiagnosed molecular origin and an exhaustive non‐diagnostic evaluation, including both ES and GS. Informed consent had been obtained from the participant's parent to participate in the NIH‐UDN protocol (15‐HG‐0130).

### 2.1 Clinical history

A 4‐year‐old male was accepted to the UDN due to refractory epilepsy, severe hypotonia, cortical visual impairment and severe global developmental delay. Seizures began at age one month and consisted of infantile spasms and tonic–clonic seizures. Treatment with ACTH resulted in cessation of seizures for one month, with a relapse once ACTH was discontinued. Since then, the seizures have been refractory to numerous antiepileptic drugs (AEDs), and continue to occur multiple times daily, despite AED polytherapy and vagal nerve stimulator implantation. The family history was non‐contributory; there was a healthy older brother. MRI brain at age three years demonstrated diffuse brain parenchymal volume loss and confluent T2 white matter signal abnormality with posterior cerebral predominance. Extensive metabolic and genetic testing prior to the UDN had been non‐diagnostic, including chromosome analysis, CMA (Affymetrix Cytoscan HD array), an infantile epilepsy panel in 2013 (sequencing and deletion/duplication analysis of 38 genes; Illumina HiSeq sequencing with analysis performed using Agilent Genomic Workbench software), mitochondrial genome sequencing and trio ES (Illumina HiSeq 2000, 130x mean depth of coverage) in 2014.

At the time of the UDN evaluation, the participant had demonstrated little developmental progress; he was unable to hold his head up without support, could not sit independently or grasp objects with his hands. He was able to make sounds but had no speech. On exam, dysmorphic features including coarse facial features, anteverted nares, and thick, prominent lips with tongue protrusion were noted. The participant also had growth delay (height at the third percentile); weight and head circumference at the 25th percentile; soft, doughy skin; hirsutism; severe, diffuse hypotonia; mild lower extremity hemihypertrophy; and small hands and feet. A *de novo* variant of unknown significance (NM_012292.4:c.838G>C, p.Gly280Arg) in *ARHGAP45* had been reported on ES by the commercial laboratory. However, *ARHGAP45* had no known disease association, the c.838G>C variant was observed in five alleles in gnomAD, and case matching efforts through GeneMatcher were unsuccessful. Research reanalysis of the ES data through the UDN utilizing a phenotypic agnostic pipeline was non‐diagnostic and did not reveal additional reportable variants. Trio GS (Illumina HiSeq X, 43.8x mean depth of coverage) was completely negative, no variants were reported.

### 2.2 Optical genome mapping

A fresh blood sample in EDTA was collected from the participant and both parents for OGM. Collected blood was processed with the manufacturer's guidelines for the extraction of ultra‐high molecular weight (UHMW) DNA (Bionano Genomics). DNA labeling was performed following the manufacturer's protocols (Bionano Genomics). The fluorescently labeled DNA molecules were imaged sequentially across nanochannels on a Saphyr instrument. Effective genome coverage of greater than 80X was achieved for all samples.

*De novo* genome assembly, variant calling and analysis were performed using software solutions provided by Bionano Genomics. SVs were identified based on the alignment profiles between the *de novo* assembled genome maps and the Human Genome Reference Consortium GRCh38 assembly. If the assembled map did not align contiguously to the reference, but instead was punctuated by internal alignment gaps (outlier) or end alignment gaps (endoutlier), then a putative SV was identified. Targeted rare variant analyses were performed to estimate the percentage of mosaic levels for identified SVs. Briefly, mosaic variant frequencies were determined by a custom workflow. First, the raw molecules were realigned to all consensus genome maps and the reference GRCh38, then the allele frequency of a variant was computed by dividing the variant‐allele coverage on the consensus map by the coverage on the reference assembly and all other maps in the vicinity. SVs were annotated with the combination of Variant Annotation Software (Bionano Genomics) and an open‐source R package, *nanotatoR* (Bhattacharya et al., 2021). Analysis was performed using the *nanotatoR’s* output and images were generated with Bionano Access (Bionano Genomics). The OGM base‐pair resolution is limited due to utilization of label patterns and measurement of distances between them. This means that the actual breakpoint could be anywhere between the two labels showing SVs. On average this should be approximately 3 kb, but could be as large as 10 kb. Confirmation of the deletion and refinement of the breakpoints in a CLIA‐certified laboratory was obtained via mate‐pair sequencing at Mayo Clinic Laboratories.

### 2.3 Mate‐pair sequencing for orthogonal confirmation in a CLIA‐certified laboratory

DNA was extracted from a peripheral blood specimen using the PureGene protocol (Qiagen), and 1 ug was utilized for mate‐pair sequencing (MPseq). Library preparation was performed using the Illumina Nextera Mate Pair library kit (Illumina). Library preparation consisted of cleavage and tagging of DNA, strand displacement to fill any gaps, and overnight circularization (16–20 hours) to produce stabile 2–5 kb DNA fragments. Ampure purification (Beckman Coulter, Indianapolis, IN) was performed after the tagmentation and strand displacement steps (0.56X and 0.4X, respectively) to ensure the selection of only the longest fragments. After circularization, non‐circularized DNA was digested with exonuclease prior to mechanical shearing of the circularized fragments with a Covaris LE220 System (Covaris). The resulting biotinylated DNA fragments were bound to Dynabeads M‐280 Streptavidin (Thermo Fisher Scientific) and subsequently processed through end repair, A‐tailing, ligation of 7 bp Illumina adapters (a component of the TruSeq DNA library prep kit), and PCR using the PCR Primer Cocktail (Illumina) and KAPA HiFi HotStart Ready Mix PCR Kit (KAPA Biosystems). A 0.67X Ampure purification was performed to complete library preparation. MPseq libraries were multiplexed at two samples per lane, and paired‐end sequencing (101 bp) was then performed on the Illumina HiSeq 2500. The average bridged coverage was 47X. Data were aligned to the reference genome (GRCh38) using BIMAv3, and abnormalities were identified and visualized using SVAtools and Ingenium, which are in‐house developed tools (Drucker et al., 2014; Johnson et al., 2018; Smadbeck et al., 2018). Findings were confirmed, and breakpoints defined to single basepair resolution, using PCR with primer pairs 5’‐GCTTAAAGGTTGAGTTAGACTTCTTCC‐3’/5’‐ GCAGAGCTATTGTGGAATAAATGAGAC‐3’ and 5’‐ CCTTAGTTAAATCTGCTGGGTATATGC‐3’/ 5’‐CCCCTGAAATGAACTCTCCTTAGTTAA‐3’, followed by Sanger sequencing with the same primers.

## 3 RESULTS

Optical genome mapping resulted in a diagnosis of X‐linked dominant early infantile epileptic encephalopathy‐2 (MIM# 300672) in the UDN participant, the details of which are outlined below. Utilizing the UDN diagnosis coding tool, the diagnosis was classified as certain (Splinter et al., 2018).

### 3.1 Optical genome mapping

**FIGURE 1:**
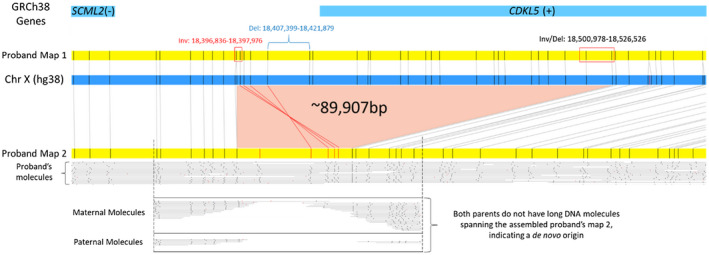
Pathogenic SV identified by Optical Genome Mapping. Reference genome GRCh38/hg38 is *in silico* digested with Bionano direct labeling enzyme 1 (DLE1) recognition sites and represented as a blue rectangular box, where black vertical lines indicate the locations of DLE1 labels. Similarly, sample maps are represented in yellow. The alignments between sample maps (yellow) and the reference genome (blue) are shown with grey lines. The highlighted middle section (light red) shows the deleted region from the reference, not present in the proband's map 2 (GRCh38, chrX: Inversion left breakpoint: 18,396,836–18,397,976; Deletion left breakpoint: 18,407,399–18,421,879; Inversion/Deletion right breakpoint: 18,500,978–18,526,526). Reference genes (light blue) are shown on top with the orientation of gene transcription (+ or – strand) shown in parenthesis. The red‐colored DLE1 label locations on proband map 2 indicate unaligned labels with the reference. These labels were manually aligned (red lines) to the reference and show a region that is inverted. The bottom two rectangular boxes contain maternal and paternal molecules aligned to the proband's map 2. No parental molecules are identified to span the area with the SVs indicating a *de novo* origin

OGM performed on the trio identified a novel (not seen in the Database of Genomic Variants or Bionano Healthy Control SV database) *de novo* 90 kb deletion on the X chromosome overlapping one gene, *CDKL5* (MIM# 300203, Figure 1). Two consensus genome maps were assembled for the participant's single X chromosome (46,XY), one normal (Proband Map 1, Figure 1) and the second (Proband Map 2) carrying the deletion, suggesting a mosaic nature of the identified variant. Investigation of the single‐molecule alignments to the reference genome determined the alternate allele fraction to be 23.6% (SV coverage—11 molecules/ total coverage—46.7). Manual investigation of unaligned labels on Proband map 2 (Figure 1, red) to the reference genome indicated a section of DNA material in the same region to be inverted in regard to the reference genome label pattern (Figure 1, red label alignments of Proband map 2 with the reference). Long single molecules used to assemble Proband Map 2, show that the two events are in phase and likely present within the same cells. There were no additional reportable findings. The X chromosome deletion and inversion were confirmed in a CLIA‐certified laboratory before communicating results to the participant's family.

### 3.2 Mate‐pair sequencing

**FIGURE 2:**
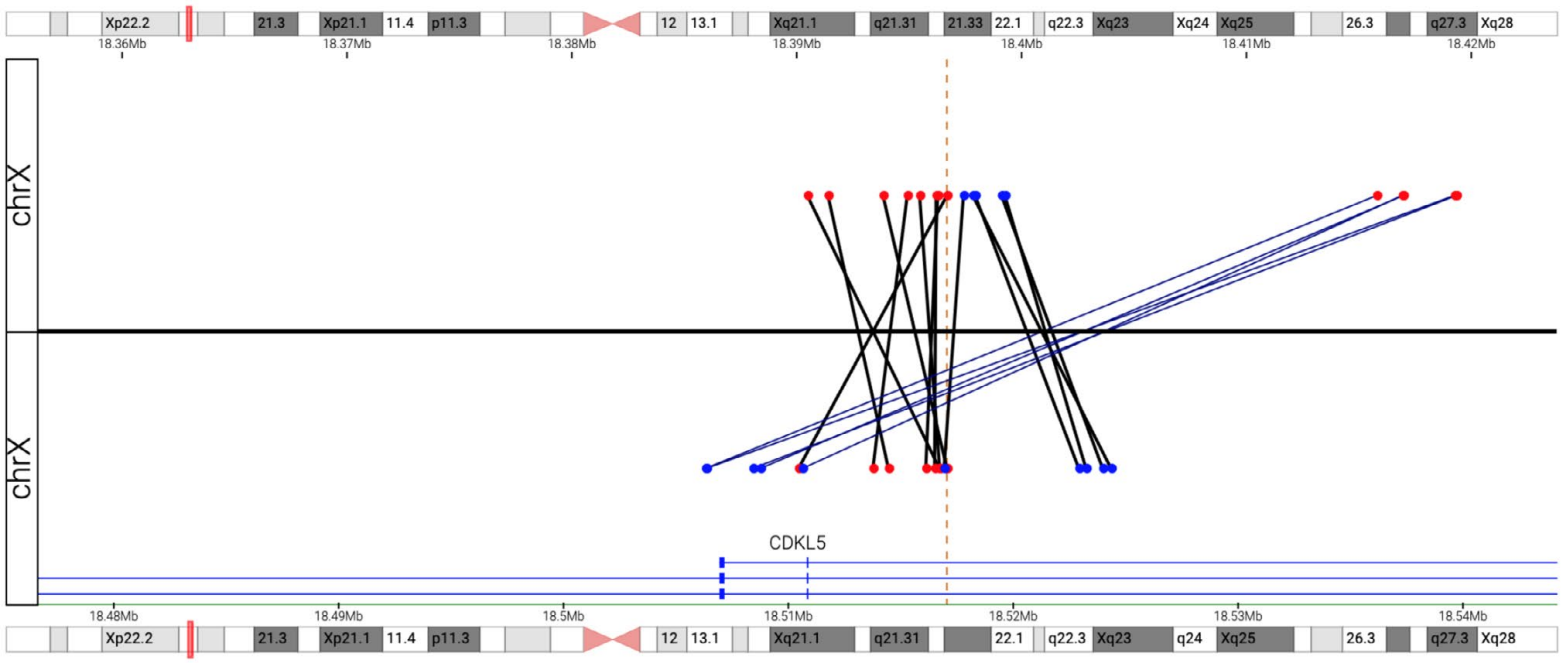
MPseq results visualized in Ingenium. The top panel represents the lower genomic coordinate of each mated pair while the bottom panel represents the higher genomic coordinate; genomic position is shown at the top and bottom in Mb. Bridged coverage of 47x for autosomes and 23.5x coverage for the X chromosome in a male patient was achieved (data not shown). The junction plot demonstrates a mosaic deletion involving the *CDKL5* gene (red→blue junctions) at chrX: 18,419,574–18,504,791. Five fragments representing the deletion were observed denoting that the deletion is mosaic (5/23.5 = 21%). Also shown are red →red and blue →blue junctions, representing an inversion in the same general region. The inversion breakpoints are located between the red and blue dots (genomic coordinates chrX:18,397,503 and chrX:18,517,260). Thirteen total mate pairs spanning the inversion breakpoints (8 covering one breakpoint, 5 covering the other, for an average of 6.5) were observed, denoting that the inversion is mosaic (6.5/23.5 = 27.7%)

MPseq identified a mosaic 85 kb deletion overlapping the *CDKL5* gene and mosaic inversion at the same location (Figure 2), estimated to be present in 21% and 27.7% of the cells, respectively. The deletion involves upstream regulatory regions as well as exons 1–3 (based on transcript NM_003159.2) of *CDKL5* and is predicted to result in functional inactivation of the gene. Of note, it is not possible to determine from the MPseq data whether the deletion and inversion co‐occur in the same cell population, though this was confirmed with the OGM data that demonstrated the deletion and inversion are present in single molecules.

## 4 DISCUSSION

Optical genome mapping detected a mosaic approximately 90 kb deletion (determined to be 85 kb by MPseq) which removes exons 1–3 of the *CDKL5* gene, representing the first use of optical genome mapping to solve an undiagnosed case within the UDN. Deficiency of *CDKL5* causes X‐linked dominant early infantile epileptic encephalopathy‐2 (MIM# 300672), also known as *CDKL5* deficiency disorder (CDD). CDD affects nearly four times as many females as males, with complete loss of function thought to be lethal in males (Fehr et al., 2013; Jakimiec et al., 2020). Mosaic SNVs in *CDKL5* have been reported in males with CDD in as few as 10% of reads (Stosser et al., 2018). A mosaic 105 kb deletion in *CDKL5*, 20 kb larger than our patient's mosaic deletion, was previously reported in a male with CDD. This 105 kb deletion was detected by CMA that included increased exon coverage of *CDKL5*, and determined by FISH to be present in 24% of cells analyzed. Bartnik et al., 2011). Our participant's genotype and phenotype are also consistent with a diagnosis of CDD, and the mosaic nature of the predicted loss of function deletion (~24% of reads) likely led to his survival (Olson et al., 2019). In addition to the deletion, OGM and MPseq detected a *de novo* inversion in the same region, providing superior SV characterization which would not have been detectable on CMA due to its balanced nature. To date, there are few reported examples of inversions predisposing to other genomic rearrangements that may lead to disease (Puig et al., 2015). There were no repetitive elements or homologies adjacent to the breakpoints that would predispose to the formation of these rearrangements.

Clinical availability of NGS, which has a higher sensitivity for the detection of mosaic SNVs and INDELs than traditional Sanger sequencing, has resulted in an increased appreciation for the contribution of mosaic variants to genetic disease (Stosser et al., 2018). An estimated 1.5% of molecular diagnoses made on ES are due to mosaic SNVs, with the ability to detect alternate allele fractions as low as 3% (average AAF 18.2%, range 3.1–79.7%; Cao et al., 2019). In males, SNVs on the X chromosome can be detected with an alternate allele fraction as low as 10% (Cao et al., 2019). Mosaic CNVs are likely more common than what is currently appreciated, but pose a particular challenge to detection as clinically available CNV detection methods have reduced sensitivity for mosaic variants. The mosaic 85 kb deletion in this participant had been missed by extensive prior genetic and genomic testing. Retrospective manual inspection of the CMA data demonstrated a suggestion of the deletion, but an 85 kb mosaic deletion outside a known microdeletion region is well below the reporting standards for CMA (Mason‐Suares et al., 2013). The prior infantile epilepsy panel had included exon‐level array CGH for *CDKL5*, however, mosaic events may not be detected by exon aCGH. Deletions involving three exons are generally amenable to detection by CNV analysis of NGS data (Retterer et al., 2015), however, in this participant, only two exons were covered on ES (Figure S1, *CDKL5* exon 1 is untranslated and was not included in the exome capture kit). Mosaic intragenic CNVs are difficult to routinely detect on ES or GS. GS in particular has improved sensitivity over ES in detecting most types of constitutional CNVs, but the lower number of reads makes identification of mosaicism challenging. Retrospective manual inspection of the GS data demonstrated evidence of the deletion in the participant in the form of coverage drop (Figure S2).

In conclusion, this participant demonstrates that the complete spectrum of pathogenic SVs is not comprehensively ascertained with currently available clinical testing. OGM has been shown to reliably detect most types of SVs with >95% sensitivity (true positives), notably with the ability to detect low‐level mosaicism starting at 5% alternate allele fraction (Genomics, 2020). While OGM is an emerging technology that requires further validation before wide‐spread use in the clinical realm, it has the potential to be a valuable diagnostic tool. The development of OGM represents a new opportunity to resolve undiagnosed diseases and further research into the utility of OGM in undiagnosed populations is warranted.

## CONFLICT OF INTEREST

HB and EV own a limited number of stock options of Bionano Genomics Inc. HB is also employed part‐time by Bionano Genomics Inc. All other authors declare no conflict of interest.

## AUTHOR CONTRIBUTIONS

HC, NW and VS participated in the patient's UDN evaluation and collected the clinical data. HB, SB, YF and EV performed optical genome mapping and analysis. NH and CM performed mate‐pair sequencing and analysis. CR and KD performed microarray and analysis. AA performed a reanalysis of ES and GS data. HC drafted the manuscript. HB and NH contributed to the writing and revision of the manuscript. SB, YF, NH, CM, NW, CR, KD, AA, EV and VA edited the manuscript. All authors approved the final manuscript.

## Supporting information

Fig S1‐S2

## Data Availability

The CNV reported herein has been submitted to ClinVar (SCV001450925). Genomic data is available at dbGaP and upon reasonable request.
