# Supplementary material for: Detection of a mosaic CDKL5 deletion and inversion by optical genome mapping ends an exhaustive diagnostic odyssey
Source: Mol Genet Genomic Med. 2021 May 6;9(7):e1665. doi: 10.1002/mgg3.1665 (PMC8372083; doi:10.1002/mgg3.1665)
Supplement: Supplementary file 1 — Fig S1‐S2 [file MGG3-9-e1665-s001.docx]

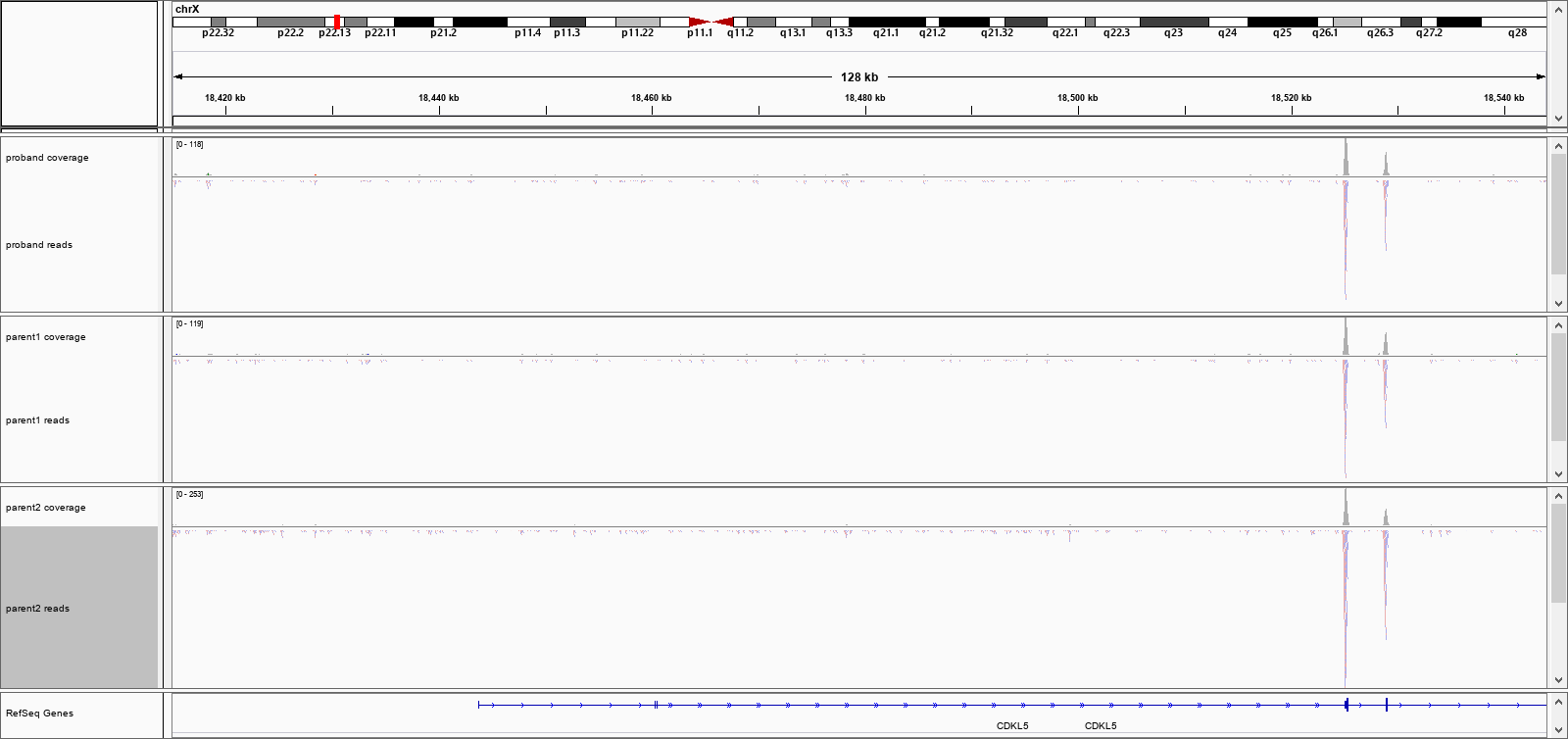


Supplementary Figure 1: Integrated Genomics Viewer (IGV) screenshot of a mosaic deletion region at Xp22.13 in the child (top) and both parents (reference genome GRCh37/hg19, chrX:18414956 – 18544646). Visualization of exome sequencing data.


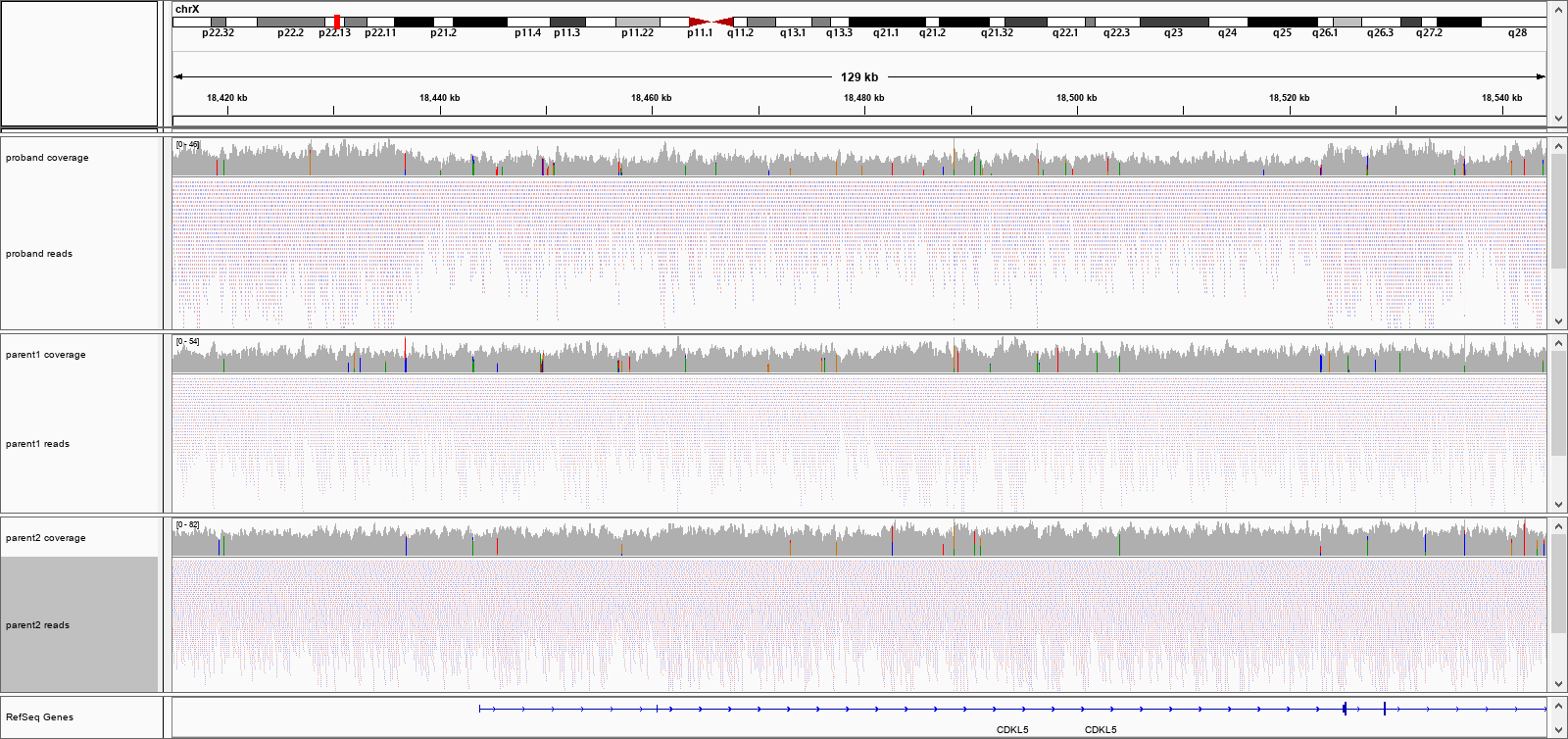


Supplementary Figure 2: Integrated Genomics Viewer (IGV) screenshot of a mosaic deletion region at Xp22.13 in the child (top) and both parents (reference genome GRCh37/hg19, chrX:18414956 – 18544646). Visualization of genome sequencing data. Evidence for the variant, in the form of coverage drop appears in the child (top).
